# A Handbook of Medical Climatology

**Published:** 1899-01

**Authors:** 


A Handbook of Medical Climatology. Embodying its Principles and Therapeutic Application with Data of the Chief Health Resorts of the World. By S. Edwin Sally, M. D., M. R. C. S., Late President of the American Climatological Association. Illustrated in Black and Colors. Published by Lea Bros. & Co., Philadelphia. 1897. Pages, 470.

Under the stimulation of governmental weather bureaus and climatological surveys, scientific men all over the world have given themselves to a study of climates, weather, water, breezes, relative humidity, rainfall, temperature and the other phenomena that go to make the science of meteorology. Physiologists have worked out their part of the problem fairly well, and we know with reasonable certainty the effects of certain meteorological phenomena upon the human organism in health and disease. The therapeutist has therefore, a new weapon placed in his hand with which to fight disease, and should not be slow to avail himself of it. Not in the old empiric way, however, of sending our syphilitics and rheumatics to the Hot Springs of Arkansas because we have heard that they were "good for" these diseases, but we should seek to make our prescription as exact and scientific, when we are prescribing climate, as when we are prescribing drugs. It is only by keeping step with the march of science and availing himself of every opportunity it offers, that the general practitioner can maintain his leadership in society, and the profession, to which he is justly entitled. The surgeon and specialists are not slow to grasp every opportunity that an unfolding science offers and we should be no less enterprising.

The work under consideration is a very valuable and a very practical one and presents the science in a clear and comprehensive manner. It is divided into three sections. The first section is devoted to a general consideration of the science of climatology. The second section is devoted to the study of therapeutics in connection with disease, and the third treats of special climates as typified by selected resorts. Much useful statistical information is included.

